# St. Mary's Hospital Medical School

**Published:** 1916-02-26

**Authors:** 


### PATRIOTISM IN BRITISH HOSPITALS.

### IV. St. Mary's Hospital Medical School.

St. Mary's has given freely with both hands to the country. Like other hospitals, it has surrendered a large number of beds to the wounded and has suffered severe depletion in its resident, teaching, and consultant staffs. In addition to these patriotic services St. Mary's laboratories have rendered most valuable aid in the battle against disease and death in many parts of the world.

### St. Mary's Men in the War.

An analysis of the 356 St. Mary's men who are serving with the Forces shows that in the Navy they have 12 fleet surgeons, 12 staff surgeons, 11 surgeons, and 23 temporary surgeons. In the Royal Army Medical Corps, including Staff appointments and those who hold temporary commissions during the war, are 7 colonels, 11 lieutenant-colonels, 24 majors, 23 captains, 120 lieutenants. In the Indian Medical Service are 1 colonel, 8 lieutenant-colonels, 18 majors, 8 captains, 2 lieutenants. In the Territorial Service are 60 commissioned officers of various ranks, while in the combatant Forces are 3 lieutenants, 1 sergeant-major, and 12 privates.

The staff has been sadly depleted, and the work of carrying on, though efficiently performed, entails a steady pressure of extra work on those who remain behind. The departure *en bloc* of the resident staff in the early days of the war was a shock from which the hospital recovered with a speed which testified to fine organisation. The senior students grappled with the work in a spirit which has reflected high credit upon them and on their *Alma Mater*.

### List of Honours.

Among distinguished members of the St. Mary's School Sir Almroth Wright comes first, mentioned in dispatches and recipient of the C.B. The C.M.G. has been awarded to Lieutenant-Colonel T. H. J. C. Goodwin, Lieutenant-Colonel C. H. Hale, D.S.O., and Lieutenant-Colonel G. T. K. Maurice, also mentioned in dispatches. The D.S.O. has been awarded to Surgeon-Major Basil Pares; Majors R. V. Cowey, P. G. Easton, and Nelson Low, all of whom were mentioned in dispatches; and to Captain A. Graham Butler. The Military Cross has been awarded to Captains J. E. M. Boyd, A. G. W. Compton, and J. R. M. Whigham, all of whom have been mentioned in dispatches. The following have also been mentioned in dispatches: Colonel E. O. Wight (Territorial), Fleet-Surgeon E. J. Finch, R.N.; Lieutenant-Colonels H. J. K. Bamfield, I.M.S., H. O. B. Bourne-Mason, R.A.M.C., and G. E. Hale, D.S.O., R.A.M.C.; Majors F. C. Lambert, E. G. Anthonisz, A. H. McN. Mitchell, J. M. B. Rahilly, and R. K. White; Temporary-Major E. L. Gowlland; Captains A. H. Bond, Alan G. Wells, H. S. Hollis (Territorial), and R. G. Knowles, I.M.S.; Temporary-Lieutenants D. Le Bas, A. G. H. Lovell, R. F. Wilkinson, and C. M. Wilson, all of the R.A.M.C.; also Surgeon-Captains E. J. H. Luxmore, 2nd Life Guards; E. D. Anderson, 1st Life Guards; Lieutenant-Colonel V. Salisbury Shappe, R.A.M.C., T.F.; and Staff Sergeant-Major H. J. Mayes, A.S.C., T.F.

### Roll of Honour.

The following St. Mary's men have fallen in the service of their country:

Colonel E. O. Wight, Assistant Director of Medical Services, who was killed in Flanders on December 19. Colonel N. Manders, A.M.S., killed in action at Gallipoli. Captain Stephen Field, taken prisoner at the battle of Le Cateau; died of typhus at Wittenberg. Captain W. F. Higginson, Adjutant, Dublin Fusiliers, a former student, killed in action at the Dardanelles. Captain G. C. Mathison, Australian Medical Service, died of wounds received at Krithia, Gallipoli. Lieutenant J. F. O'Connell, R.A.M.C., killed in action at the battle of the Aisne. Temporary-Lieutenant Ernest Stratford, R.A.M.C., died of wound received at the battle of Neuve Chapelle. Lieutenant H. Chell, 8th Battalion Royal Fusiliers, killed while attempting to rescue a comrade in the trenches in France. Surgeon F. W. Quirke, R.N., killed by explosion on board H.M.S. *Princess Irene*. Corporal H. J. Tootal, who enlisted as a private in the Wilts Regiment, killed in action at the battle of Neuve Chapelle. Temporary-Captain A. E. Bullock, L.M.S.S.A., attached 4th Middlesex Regiment, mentioned in dispatches, killed in action September 27, 1915, while attending to the wounded upon the field. Of him it is recorded by one who worked with him under fire, "He was the bravest soldier ever seen on a battlefield." Temporary-Lieutenant Edward J. Nangle, R.A.M.C., attached 1st Loyal North Lancashire Regiment, killed in action during the advance in the last days of September 1915. "The regiment was held up for a little time under fire, and Nangle, seeing a man in pain, went up to give morphia. While attending to some others he was shot through the head by a sniper." Lieutenant E. F. Gillett, R.F.A., entered as a student at St. Mary's in 1912. He enlisted at once in the R.A.M.C. at the outbreak of the war, subsequently obtained a commission in the Royal Field Artillery, and was killed while helping a wounded man. He was only twenty-one at the time of his death. Major O. R. Ennion; Captains C. T. Edmunds, A. G. W. Compton, F. F. Wilkinson, V. C. Martyn, H. S. Hollis, H. H. B. Cunningham, T. W. S. Hills, all of the R.A.M.C.; and Captain R. Knowles, I.M.S., have been wounded.

